# A Survival Case of Super-refractory Status Epilepticus due to Glutamic Acid Decarboxylase Antibodies-associated Limbic Encephalitis

**DOI:** 10.7759/cureus.3125

**Published:** 2018-08-10

**Authors:** Baoqiong Liu, Yan Zhou, Lingbin Meng, Holly Skinner

**Affiliations:** 1 Internal Medicine Residency, Florida Hospital, Orlando, USA; 2 Critical Care, Medical College In Wisconsin, Milwaukee, USA; 3 Neurology, Florida Hospital, Orlando, USA

**Keywords:** limbic encephalitis, glutamic acid decarboxylase antibodies, refractory seizures, immunotherapy

## Abstract

Limbic encephalitis (LE) is a neurological syndrome that mainly affects mesial temporal lobes. It may present in association with cancer or infection. Limbic encephalitis associated with glutamic acid decarboxylase antibodies (anti-GAD) is rare. Here, we report a case of anti-GAD limbic encephalitis to heighten the awareness of this rare cause of autoimmune encephalitis. Anti-GAD-associated epilepsy is often poorly responsive to seizure medications. Treatment is challenging. Early initiation of immunotherapy is important.

## Introduction

Glutamic acid decarboxylase (GAD) is the rate-limiting enzyme in producing gamma-aminobutyric acid (GABA), the main inhibitory neurotransmitter. GAD directed antibody (anti-GAD) is a rare cause of autoimmune limbic encephalitis, with a prevalence of 1.9/100,000. It mainly affects mesial temporal lobes [1, 2]. Patients usually present with altered mental status, cognitive impairment and refractory seizures [3, 4]. We report a case of anti-GAD-associated limbic encephalitis, presenting with super-refractory status epilepticus. Informed consent statement was obtained for this study.

## Case presentation

A 41-year-old previously healthy Korean man presented with fever and headache for four days, and altered mental status for one day. The family had difficulty waking him up and he was "picking things out of the air". He had no past medical history or any family history of autoimmune diseases. Upon arrival, he was lethargic but without a focal neurologic deficit and had a fever of 100.6°F. Remarkable labs included white blood cell (WBC) count of 3.55 x 10^9^/L with a bandemia of 20%. Routine cerebral spinal fluid (CSF) study results showed elevated WBC count of 72/ml, red blood cell count (RBC) of 24/ml, and protein of 118 mg/dl. CSF glucose was within normal limit at 70 mg/dl. He was empirically treated with vancomycin, ceftriaxone, acyclovir, and dexamethasone. However, his mental status worsened quickly and required intubation.

**Figure 1:**
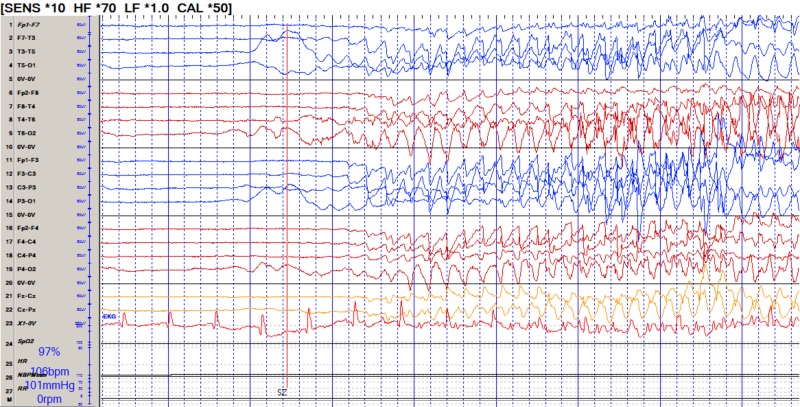
Continuous electroencephalogram (EEG) showing breakthrough seizures in spite of pentobarbital-induced burst suppression.

**Figure 2:**
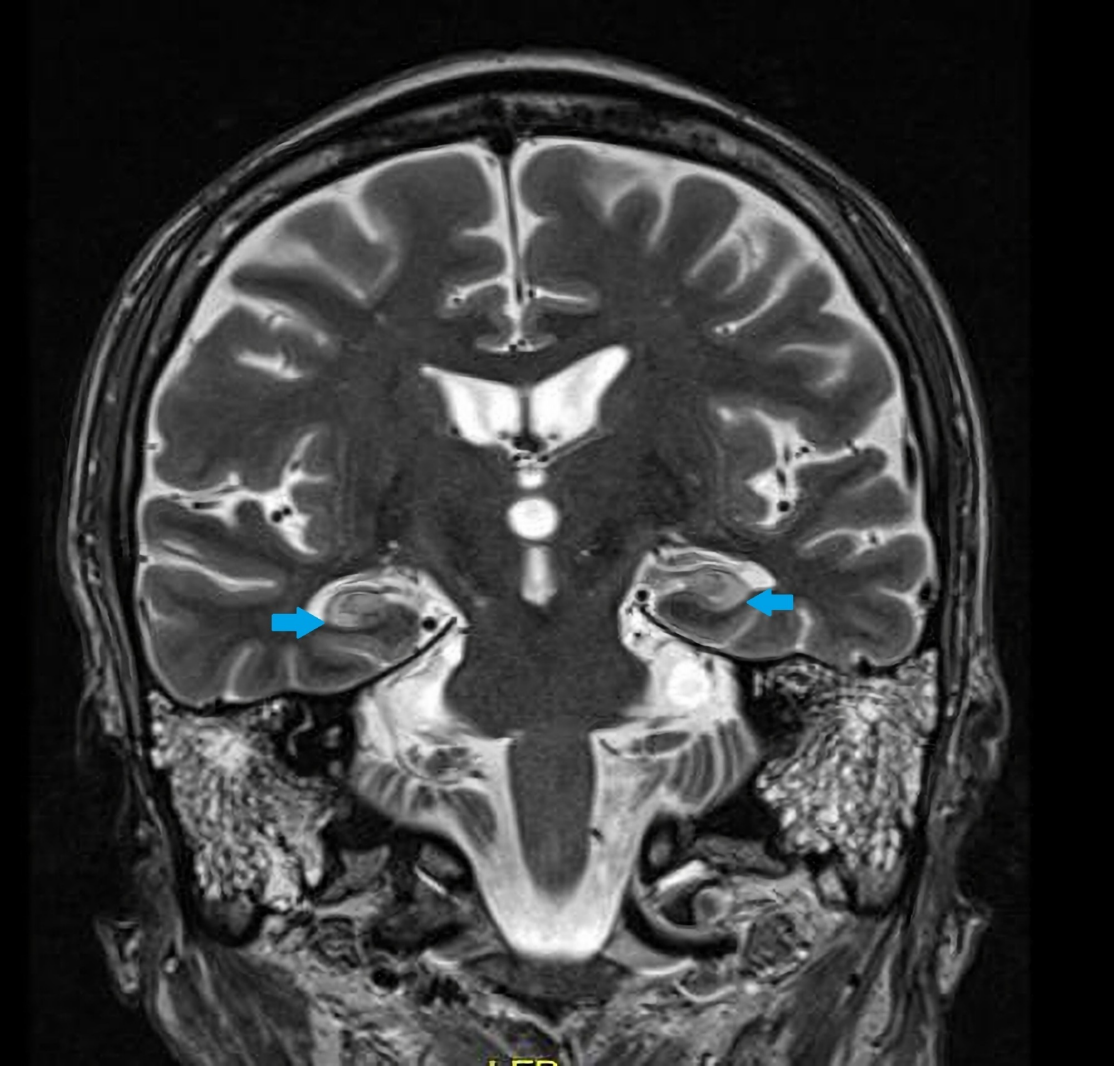
Magnetic resonance imaging (MRI) brain coronal T2 image demonstrating increased T2 signal in the bilateral hippocampi (blue arrows).

He was placed on continuous video electroencephalogram (EEG) and found to be in non-convulsive status epilepticus (NCSE) (Figure 1). Extensive infectious workup and cancer screening, including a whole body computed tomography (CT), testicular ultrasound, and flow cytometry of peripheral blood were negative. However, autoimmune workup was remarkable for elevated anti-GAD of >250 u/ml and antinuclear antibody (ANA) titer 1:320. Magnetic resonance imaging (MRI) brain demonstrated increased signal in the bilateral mesial temporal lobes (Figure 2).

NCSE continued despite pentobarbital-induced burst suppression necessitating the addition of midazolam and ketamine drips with multiple failed attempts to wean off these sedative-hypnotic medications. All other available intravenous seizure medications (Phenytoin, valproic acid, levetiracetam, phenobarbital and lacosamide) were utilized in various combinations while trying to wean sedative-hypnotic drips. Besides, the patient received a ketogenic diet.

Diagnosis of anti-GAD-associated autoimmune encephalitis was made based on the clinical course and workup. Immune targeted therapies began with high dose intravenous steroids, then intravenous immunoglobulin (IVIG). Next, he was treated with plasmapheresis which allowed for improvement of seizures activity, tapering of sedative-hypnotic medications and regaining consciousness. However, frequent intermittent seizures continued despite the use of multiple seizure medications. Thus, additional immunotherapies were given. Anakinra (an interleukin 1 receptor antagonist) and Mycophenolic acid were also added.

Three months later, he was decannulated. He improved to be alert and oriented to person and place, with intelligible speech, memory impairment, and mild generalized weakness. Short-term seizure control was achieved using with five seizure medications including oxcarbazepine, phenobarbital, lorazepam, clonazepam, and perampanel. Anti-GAD level was decreased to 17.6 u/ml at the time of discharge.

## Discussion

GAD antibody has been reported to be in association with both paraneoplastic [5-6] and nonparaneoplastic [7] autoimmune encephalitis. Anti-GAD limbic encephalitis is challenging to diagnose as anti-GAD is not always included in the typical paraneoplastic/autoimmune panels. In patients with anti-GAD limbic encephalitis, the CSF anti-GAD antibody titers are often lower than that in the serum. EEG is usually nonspecific. MRI T2-weighted hyperintensity and "swelling" in mesial temporal structure can be found in the acute/subacute phase [6, 7]. For patients with a suspected paraneoplastic syndrome, workup including a whole body CT or a positron emission tomography (PET) scan can be performed to look for tumors. Bone marrow biopsy may also be considered if lymphoma is suspected [8]. In our case, anti-GAD-associated autoimmune encephalitis appears to be nonparaneoplastic, as a whole body CT scan, testicular ultrasound, and flow cytometry are negative.

Due to the relative rarity of the disease, there are no prospective trials in this patient population to guide management. All available experience is from case reports. Anti-GAD-associated epilepsy is often poorly responsive to seizure medications [9]. The goal is to reduce immune response and enhance GABAergic activity. Unlike the other autoimmune encephalitis, anti-GAD encephalitis is very resistant to immunotherapy [10]. The non-convulsive status epilepticus of our patient was not well controlled until he received intravenous steroids, IVIG, and subsequent plasmapheresis. Besides, early initiation of immunotherapy should be undertaken before the pathological effects spread to extra-temporal areas which can make the treatment even more challenging.

## Conclusions

Anti-GAD limbic encephalitis is a challenging condition to diagnose and treat. It is not always included in the typical paraneoplastic/autoimmune panels. Anti-GAD-associated epilepsy is often poorly responsive to anti-epileptic drugs. Unlike the other autoimmune encephalitis, anti-GAD encephalitis is relatively resistant to immunotherapy. Immunosuppressants beyond conventional treatments such as intravenous immunoglobulin and plasmapheresis may be necessary and further research is needed to better understand which treatments work best.
